# Identification of Variety and Prediction of Chemical Composition in Cocoa Beans (*Theobroma cacao* L.) by FT-MIR Spectroscopy and Chemometrics

**DOI:** 10.3390/foods12224144

**Published:** 2023-11-16

**Authors:** Lucero Azusena Castillejos-Mijangos, Ofelia Gabriela Meza-Márquez, Guillermo Osorio-Revilla, Cristian Jiménez-Martínez, Tzayhri Gallardo-Velázquez

**Affiliations:** 1 Departamento de Ingeniería Bioquímica, Instituto Politécnico Nacional, Escuela Nacional de Ciencias Biológicas-Zacatenco, Av. Wilfrido Massieu s/n, Esq. Cda. Miguel Stampa, Col. Unidad Profesional Adolfo López Mateos, Zacatenco, Alcaldía Gustavo A. Madero, Ciudad de México C.P. 07738, Mexico; castillejos767@gmail.com (L.A.C.-M.); ogmmz@yahoo.com.mx (O.G.M.-M.); osorgi@gmail.com (G.O.-R.); crisjm_99@yahoo.com (C.J.-M.); 2 Departamento de Biofísica, Instituto Politécnico Nacional, Escuela Nacional de Ciencias Biológicas-Santo Tomás, Prolongación de Carpio y Plan de Ayala s/n, Col. Santo Tomás, Alcaldía Miguel Hidalgo, Ciudad de México C.P. 11340, Mexico

**Keywords:** cocoa, FT-MIR spectroscopy, chemometric, SIMCA model, PLS algorithm

## Abstract

Cocoa is rich in polyphenols and alkaloids that act as antioxidants, anticarcinogens, and anti-inflammatories. Analytical methods commonly used to determine the proximal chemical composition of cocoa, total phenols, and antioxidant capacity are laborious, costly, and destructive. It is important to develop fast, simple, and inexpensive methods to facilitate their evaluation. Chemometric models were developed to identify the variety and predict the chemical composition (moisture, protein, fat, ash, pH, acidity, and phenolic compounds) and antioxidant capacity (ABTS and DPPH) of three cocoa varieties. SIMCA model showed 99% reliability. Quantitative models were developed using the PLS algorithm and favorable statistical results were obtained for all models: 0.93 < R^2^c < 0.98 (R^2^c: calibration determination coefficient); 0.03 < SEC < 4.34 (SEC: standard error of calibration). Independent validation of the quantitative models confirmed their good predictive ability: 0.93 < R^2^v < 0.97 (R^2^v: validation determination coefficient); 0.04 < SEP < 3.59 (SEP: standard error of prediction); 0.08 < % error < 10.35). SIMCA model and quantitative models were applied to five external cocoa samples, obtaining their chemical composition using only 100 mg of sample in less than 15 min. FT-MIR spectroscopy coupled with chemometrics is a viable alternative to conventional methods for quality control of cocoa beans without using reagents, and with the minimum sample preparation and quantity.

## 1. Introduction

Cocoa (*Theobroma cacao* L.) is a species native to the humid tropics of America, especially Mesoamerica [1]. For the peoples of Mesoamerica, it was considered a gift from the gods; the fruit symbolized the human heart and was ceremonially used in royal weddings, military victories, or the successful conclusion of commercial expeditions [2].

*T. cacao* L. is a raw material for the chocolate, beverage, and confectionery industries. Additionally, it is a source of polyphenols and alkaloids (theobromine and caffeine) that act as antioxidants, anticancer agents, and anti-inflammatory compounds, protecting the human body against cardiovascular diseases and diabetes [3,4,5].

The crops of *T. cacao* L. are classified into three different varieties: criollo, forastero, and trinitario, each of which has specific sensory and physicochemical characteristics that, along with the fermentation and drying process, determine its quality [2].

Cocoa contains, on a dry basis, approximately 16% proteins, 50% lipids (cocoa butter), and 20% carbohydrates, in addition to alkaloids such as theobromine (0.8–1.4%) and caffeine (0.1–0.7%). These latter compounds are used as criteria to differentiate between fine and bulk cocoa, thus ensuring authenticity, quality, and price [6,7].

The analytical methods commonly used to quantify the proximate chemical composition of cocoa, phenolic compounds, and antioxidant capacity are laborious, expensive, slow, and detrimental to both the analyst and the environment due to the use of chemical reagents and solvents. This indicates that rapid, simple, cost-effective, and environmentally friendly analytical methods should be developed to facilitate the evaluation of cocoa quality [7,8]. FT-MIR spectroscopy has proven to be an excellent alternative to conventional methods, and when combined with multivariate analysis, it has been used in many food matrices [9].

Several studies have been reported to predict the chemical composition and quality of cocoa using FT-MIR and FT-NIR. Veselá et al. [10] predicted the chemical composition of cocoa powder. Aculey et al. [11] studied changes in cocoa beans during fermentation. Teye et al. [12] estimated the quality category of cocoa beans based on their degree of fermentation (unfermented, partially fermented, and fermented). Batista et al. [13] predicted the total content of phenolic compounds and antioxidant capacity in cocoa beans with different types of fermentation. Finally, Hashimoto et al. [7] developed prediction models for quality control of cocoa beans.

To date, there have been no reported studies utilizing FT-MIR spectroscopy coupled with multivariate analysis to identify varieties of *T. cacao* L. (criollo, forastero, and trinitario). There are also no studies available for predicting the chemical composition (moisture, protein, fat, ash, pH, acidity, and phenolic compounds) and antioxidant capacity of cocoa beans.

The present study aimed to develop chemometric models based on FT-MIR spectroscopy to: (i) identify varieties of dried fermented cocoa beans; (ii) predict the chemical composition and antioxidant capacity of dried fermented cocoa beans, because these parameters are considered crucial for evaluating cocoa quality.

## 2. Materials and Methods

### 2.1. Reagents

All reagents were of analytical grade. The following reagents were acquired from Sigma Aldrich Chemical Co. (St. Louis, MO, USA): Folin–Ciocalteu reagent, 1,1-Diphenyl-2-picrylhydrazyl (DPPH), 2,20-Azino-bis (3-ethylbenzthiazoline-6-sulfonic acid) (ABTS), and 6-Hydroxy-2,5,7,8-tetramethylchroman-2-carboxylic acid (Trolox). Phenolphthalein, sodium hydroxide, potassium sulfate, sodium hydroxide, hydrochloric acid, hexane, methanol, sodium carbonate, potassium persulfate, ethanol, and gallic acid were acquired from J.T. Baker (Center Valley, PA, USA).

### 2.2. Samples

A total of 108 samples from three varieties (28 criollo, 40 forastero, and 40 trinitario) of dried fermented cocoa beans (*T. cacao* L.) were used. The samples were provided by the National Institute of Forestry, Agricultural, and Livestock Research (INIFAP) at the Huimanguillo experimental field (17°50′07″ N 93°23′27″ W). The forastero and trinitario cocoa variety samples were acquired from the December 2018–February 2019 harvest period, and the criollo variety cocoa samples were from the December 2020 harvest period. The cocoa beans were inspected to remove foreign matter (such as stones or defective beans), and then the samples were stored in vacuum-sealed polyethylene bags (Selovac, model 200B, São Paulo, Brazil) properly labeled. Finally, the samples were stored at room temperature in a dry place until their analysis.

### 2.3. Sample Preparation

Before conducting the proximate chemical analysis, the cocoa beans were manually husked, and their interior was inspected following the guidelines set by Mexican legislation [14]. Subsequently, the cocoa beans were crushed and frozen at −195 °C with liquid nitrogen. The samples were then ground using a food processor (Magic Bullet Deluxe, Magic Bullet, Mexico City, Mexico) for 3 cycles of 30 s each, allowing a 10-min cooling period after successive grinding to minimize the loss of volatile compounds. Next, the samples were sieved through a No. 40 mesh (425 µm) to obtain a homogeneous powder [7,12].

### 2.4. Chemical Analysis

All chemical analyses were performed in triplicate. The chemical analyses included moisture determination, which was carried out using the rapid thermobalance method [15]. Official AOAC methods [16] were followed to determine protein (method 970.22), fat (method 963.15), ash (method 923.03), pH (method 970.21), and acidity (method 942.15).

The extraction of phenolic compounds was according to Vázquez-Ovando et al. [17], with modifications: 0.5 g of ground cocoa (previously defatted) was mixed with 10 mL of a methanol-water-HCl solution (1:1:1 *v*/*v*, pH 2) and magnetically stirred for 24 h at room temperature. Subsequently, the mixture was centrifuged at 3400 rpm for 15 min, and the supernatant was collected in an amber vial and stored at −20 °C. A second extraction was performed under the same conditions, using the precipitate. After 24 h, the second supernatant was obtained and mixed with the first supernatant collected. The total extract was stored at −20 °C.

Phenolic compounds were determined according to Singleton et al. [18]. Absorbance was measured at 750 nm using a spectrophotometer (Jenway 6320D, Staffordshire, UK). The calibration curve (0–1 mg/mL, *n* = 7, R^2^ = 0.9989) was constructed using gallic acid as a standard. The results were expressed as a g gallic acid equivalent (GAE) per 100-g dry weight (g GAE/100 g dw).

Two methods (ABTS and DPPH) were used to measure antioxidant capacity. ABTS assay was performed according to the method described by Sánchez-González et al. [19]. The absorbance was measured at 734 nm (Jenway 6320D, Staffordshire, UK). The calibration curve (100–2000 µM, *n* = 5, R^2^ = 0.9994) was elaborated using Trolox as standard. The results were expressed as mmol Trolox equivalent per 100-g dry weight (mmol TE/100 g dw).

DPPH assay was performed according to the method described by Brand-Williams et al. [20]. The absorbance was measured at 515 nm (Jenway 6320D, Staffordshire, UK). The calibration curve (50–1000 µM, *n* = 6, R^2^ = 0.9999) was elaborated using Trolox as standard. The results were expressed as mmol Trolox equivalent per 100-g dry weight (mmol TE/100 g dw).

### 2.5. Statistical Analysis

The results were analyzed by descriptive statistics (mean and standard deviation), one-way analysis of variance (ANOVA), and comparison of means using the Tukey method, with a significance level of 5% (α = 0.05). The correlation between phenolic compounds and antioxidant capacity (ABTS and DPPH) was determined through Pearson correlation tests. The statistical analysis was performed with the software Minitab 18 (Minitab, Inc., State College, PA, USA).

### 2.6. FT-MIR Spectra

The infrared spectra of the cocoa samples were obtained using an FTIR spectrophotometer (model Frontier, PerkinElmer^®^, Waltham, MA, USA) equipped with a deuterated triglycine sulfate (DTGS) detector and an Attenuated Total Reflectance (ATR) accessory with a diamond crystal. The FT-MIR spectra were acquired in absorbance units (A) within the wavenumber range of 4000–550 cm^−1^, at a resolution of 4 cm^−1^ with 64 scans. Prior to sample reading, a background spectrum was collected against air under the same conditions as the samples to be used as a reference.

Approximately 30 mg of ground cocoa were placed on the diamond ATR accessory. After obtaining the spectrum of each sample, the sampling accessory was cleaned with Extran^©^ 10%, rinsed with distilled water, and dried with a soft tissue to remove any water residues. Readings were taken in triplicate, and the FT-MIR spectra were averaged using Spectrum version 10.5.3.738 software (PerkinElmer^®^, Waltham, MA, USA).

### 2.7. Multivariate Analysis

#### 2.7.1. Principal Component Analysis (PCA)

PCA was used to determine the exploratory analysis of spectra. PCA was performed using the statistical software Minitab 18 (Minitab, Inc., State College, PA, USA).

#### 2.7.2. SIMCA Model

The SIMCA model was built using 81 average FT-MIR spectra (21 criollo, 30 forastero, and 30 trinitario), which were input into Assure ID software version 4.3.8.210 (PerkinElmer^®^, Waltham, MA, USA) to form classes corresponding to the three varieties of cocoa. Subsequently, Assure ID software generated the model for discriminating between the cocoa varieties. Assure ID software incorporates the SIMCA (soft independent modeling class analogy) recognition pattern, which utilizes principal component analysis (PCA) to carry out sample classification. SIMCA model is a supervised classification method that determines whether a new sample belongs or not to a pre-existing group of samples [21].

The SIMCA model was optimized to achieve the best prediction results. The optimization involved using the following pretreatments: spectral blanks (2930–2905 cm^−1^), environmental filters (to remove CO_2_ and H_2_O), normalization (multiplicative scatter correction, MSC), Savitzky-Golay filter (9-point smoothing), and baseline correction (offset type).

The selection of the best SIMCA model was based on the following statistical parameters [21]: (i) projection of the first three principal components (PC) that demonstrate the separation or lack thereof between classes. (ii) Interclass distance, which should be greater than or equal to 3, indicating the similarity between classes. (iii) Recognition percentage and rejection percentage, both of which should be 100% if the SIMCA model correctly identified the classes.

To verify the functionality of the SIMCA model, it was validated with 27 average FT-MIR spectra (7 criollo, 10 forastero, and 10 trinitario). The FT-MIR spectra used to validate the SIMCA model were different from those used to build the model. To assess the predictive ability of the SIMCA model, the following statistical parameters were analyzed [21]: (i) total distance (should be less than 1, indicating that the sample was correctly identified), (ii) limit distance (should be equal to 1, indicating that the validated spectrum belongs to the specified population), (iii) model distance (should be equal to 0, indicating the difference in distance between the validated spectrum and the distances of the model spectra), (iv) residual distance (should be less than 3, higher values indicate that the sample contains a source of variation not previously encountered).

#### 2.7.3. Quantitative Models

The quantitative model for predicting the chemical composition of cocoa beans was constructed using 81 average FT-MIR spectra (21 criollo, 30 forastero, and 30 trinitario). These spectra were input into Spectrum Quant version 10.5.3.738 (PerkinElmer^®^, Waltham, MA, USA) along with the analytical values of the chemical composition (moisture, protein, fat, ash, pH, acidity, phenolic compounds, and antioxidant capacity). The Quant program incorporates the PLS algorithm, which correlates the FT-MIR spectra with the analytical values of chemical composition.

The quantitative model for predicting the chemical composition of cocoa beans was optimized to achieve the best prediction results. The optimization involved using the following pretreatments: spectral blanks, environmental filters (to remove CO_2_ and H_2_O), normalization (standard normal variate, SNV), Savitzky-Golay filter (9-point smoothing), and baseline correction (first and second derivative, 2 points).

The selection of the best model was based on the following statistical parameters [22]: (i) factors corresponding to the minimum value in the standard error of prediction (SEP). (ii) Calibration determination coefficient (R^2^c), which should be as close to 1 as possible and indicates the dispersion of data around the fitted straight line. (iii) Standard error of calibration (SEC), which should be as low as possible and indicates whether the model fits the calibration data or not.

To verify the predictive capability of the model, it was validated with 27 average FT-MIR spectra (7 criollo, 10 forastero, and 10 trinitario). The FT-MIR spectra used for model validation were different from those used to build the model. To assess the predictive ability of the model, the following statistical parameters were analyzed [22]: (i) validation determination coefficient (R^2^v), which should be as close to 1 as possible. (ii) Standard error of prediction (SEP), which should be as low as possible. (iii) Mahalanobis distance, which should be less than 1, indicating spectral similarity between samples. (iv) Residual ratio, which should be less than 3; if not, the sample has different characteristics from those in the model. (v) Percentage of error between actual and predicted data, which should be as low as possible.

The models were applied to five samples of cocoa beans different from those used in the calibration and validation sets. Applying the model is important for evaluating the prediction of unknown samples. Generally, the results obtained are satisfactory, making this phase a second verification of the model’s predictive capability [23].

## 3. Results

### 3.1. Chemical Analysis

The results of the chemical analysis of the three varieties of fermented dry cocoa are presented in Table 1, which coincide with the established legislation [24,25] and with other authors [17,26,27,28,29,30,31].

The three varieties of cocoa showed no statistically significant differences (*p* ≤ 0.05) in the percentage of fat, ash, and acidity. The Criollo variety showed a statistically significant difference (*p* ≤ 0.05) from the other two varieties in terms of pH value and protein. The moisture percentage only showed a significant difference between Criollo and Trinitario. These differences may be a result of the drying process carried out by the producers, which is subject to local variations depending on its duration, ranging from 7 to 12 days depending on the harvest period and local weather conditions [32]. The content of phenolic compounds and antioxidant capacity, according to both methods, showed statistically significant differences (*p* ≤ 0.05) among the three varieties of cocoa. The Forastero variety exhibited the highest content of phenols and antioxidant capacity, followed by the Trinitario variety, and finally, the Criollo variety.

The correlation between phenolic compounds and antioxidant capacity (ABTS and DPPH) was determined through Pearson correlation tests. A positive correlation was found between the phenolic content and antioxidant capacity of ABTS (r = 0.945, *p* ≤ 0.05) and DPPH (r = 0.909, *p* ≤ 0.05). This indicates that higher phenolic compound content corresponds to greater antioxidant capacity in cocoa. The above coincides with other authors [29,33,34,35,36].

Based on the chemical analysis of the three varieties of cocoa, it was not possible to differentiate between the varieties. Therefore, it is necessary to conduct a multivariate analysis to distinguish between Criollo, Forastero, and Trinitario cocoa.

### 3.2. FT-MIR Spectra

**Figure 1:**
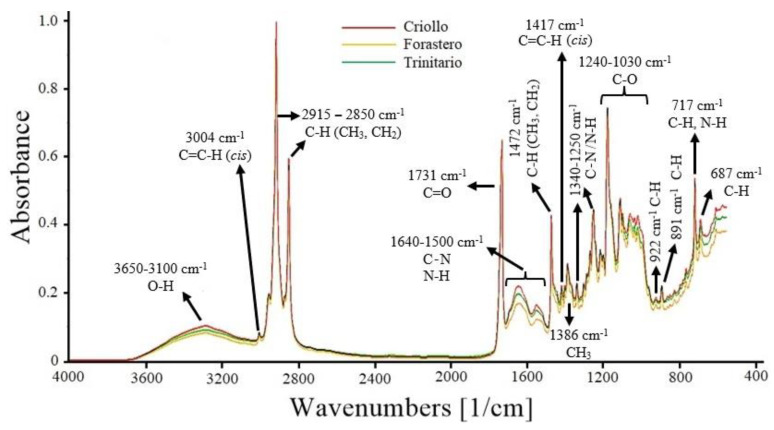
FT-MIR spectra of ground cocoa beans (Criollo, Forastero, and Trinitario).

The FT-MIR spectra of the three cocoa varieties are presented in Figure 1. The band at 3650–3100 cm^−1^ corresponds to the stretching vibrations of the O−H functional group. Other compounds, such as carbohydrates, polyphenols, and organic acids (acetic, citric, and oxalic acid) present in cocoa, also exhibit O−H stretching vibrations in the same region [37,38].

The peak at 3004 cm^−1^ is attributed to the stretching of the *cis* double bond (C=C−H) present in unsaturated fatty acids, which in cocoa is attributed to oleic acid (35% of total fat), linoleic, and linolenic acids (≤5%) [39]. The bands at 2915–2850 cm^−1^ are assigned to stretching vibrations of methyl (CH_3_) and methylene (CH_2_) C−H bonds. These bonds are found in the hydrocarbon chains of saturated fatty acids, such as stearic and palmitic acid, which make up 60% of the composition of cocoa butter [40].

The peak at 1731 cm^−1^ is associated with the C=O group of triglycerides, which is used to estimate the fat content. The range from 1640 to 1500 cm^−1^ is attributed to the C−N and N−H groups of primary and secondary amines in proteins. In this region, absorptions of C=C and C=N from pyrimidine and imidazole compounds are also present. These latter compounds are part of the structure of alkaloids (theobromine, caffeine, and theophylline) found in cocoa beans [29,37]. The peak at 1472 cm^−1^ is due to bending vibrations of methyl (CH_3_) and methylene (CH_2_) C−H bonds. The peak at 1417 cm_-1_ is attributed to the bending of the C=C−H (*cis*) bond present in unsaturated fatty acids. At 1386 cm^−1^, it corresponds to the bending vibrations of CH_3_ groups [39,41]. The region from 1340 to 1250 cm^−1^ corresponds to bending vibrations of the C−N and N−H groups of primary amines in proteins and aromatic compounds [37]. In the range from 1240 to 1030 cm^−1^, several peaks are observed, corresponding to stretching and bending vibrations of the C−O bonds in triglycerides present in cocoa beans. Approximately 98% of cocoa fat is composed of triglycerides [41,42]. Finally, the peaks between 922 cm^−1^ and 687 cm^−1^ correspond to bending vibrations of the C−H and N−H groups of various aromatic compounds present in cocoa, such as furans and pyrroles (922 cm^−1^), pyridines (891 cm^−1^), pyrazines (717 cm^−1^), and purines (687 cm^−1^) [43].

The FT-MIR spectra of the three cocoa varieties showed differences in absorbance, particularly in the fingerprint region (≤1600 cm^−1^). Therefore, some regions may be statistically different, and the application of multivariate analysis could assist in identifying the cocoa varieties.

### 3.3. Multivariate Analysis

#### 3.3.1. Principal Component Analysis (PCA)

PCA was conducted to explore—with the smallest number of principal components—the variation among the results obtained for the 108 cocoa samples (28 Criollo, 40 Forastero, 40 Trinitario).

**Figure 2:**
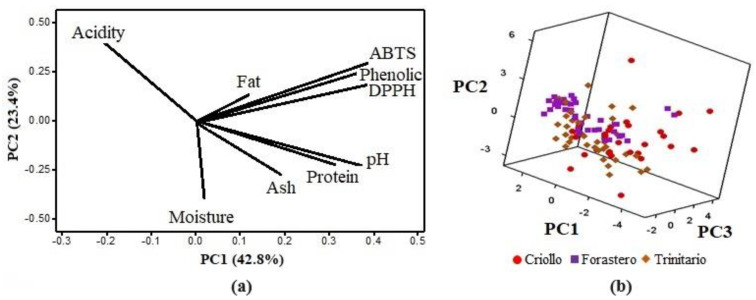
Principal component analysis. (**a**) Principal component loading plot. (**b**) Three−dimensional dispersion of PCA scores for Criollo, Forastero, and Trinitario.

Figure 2a presents the loading plot for the first two principal components, where it is observed that the first principal component (PC1) accounts for 42.8% of the total variability, while the second (PC2) accounts for 23.4%. The PCA indicated that the first four principal components are the most significant, explaining 88.3% of the variation in the samples. The variables most strongly correlated with PC1 are phenolic compounds, ABTS, and DPPH, and these variables are positively correlated, confirming the result obtained with the Pearson correlation. PC2 is positively related to acidity and negatively related to moisture and pH, meaning that high acidity values are associated with lower pH and moisture levels. PC3 accounts for 13.4% of the total variability and is positively correlated with fat content and negatively correlated with ash content. Protein content and moisture are negatively correlated in PC4, which constitutes 8.8% of the total variability.

In the 3D scatter plot (Figure 2b), the PCA scores represent the samples in a new, smaller-dimensional space. It can be observed that samples from the three cocoa varieties overlap, without clustering into defined populations. This indicates that chemical analysis alone is not sufficient to identify the cocoa varieties, and a more specific analysis, such as the SIMCA model, is required.

#### 3.3.2. SIMCA Model

**Figure 3:**
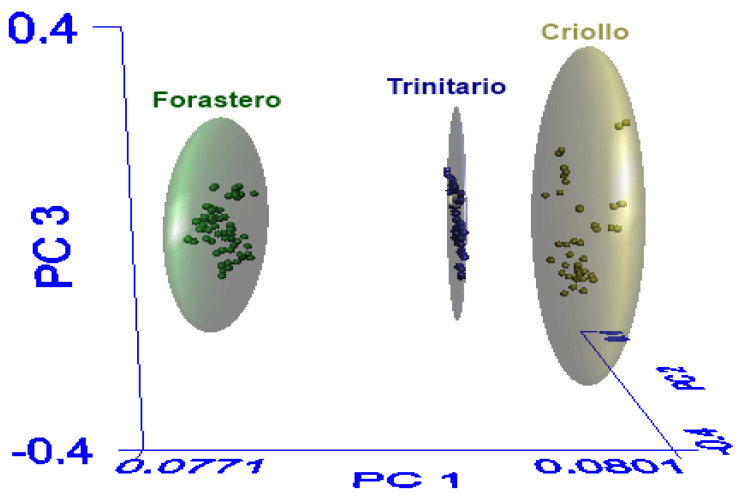
Three-dimensional principal component analysis scores plot of the populations derived from SIMCA.

Figure 3 presents the spatial distribution of the three cocoa varieties using the first three principal components, where the correct separation of classes (Criollo, Forastero, and Trinitario) is observed. The elliptical spaces (clusters) represent the 99% confidence interval that the contained samples belong to the assigned class. In the spatial distribution of the SIMCA model, it is observed that the Criollo class has a greater similarity to the Trinitario class, which coincides with the interclass distance.

The interclass distance should be equal to or greater than 3, indicating that the classes are different [21]. The interclass distance between Criollo and Trinitario was 3.34, while the interclass distance between Forastero and Trinitario was 4.01. Lastly, the distance between Forastero and Criollo was 4.29. These results coincide with studies reporting differences among cocoa varieties, indicating that Forastero cocoa is of lower quality and Criollo cocoa has a fine aroma. Therefore, the difference in the aromatic profile is more notable in these two varieties, while Trinitario cocoa, being a hybrid, falls in the medium-high quality range [28,44].

In addition to the interclass distance, another useful parameter for assessing the SIMCA model’s performance is the recognition percentage and rejection percentage. The recognition percentage (sensitivity) is the number of samples belonging to a class that are correctly recognized by the model, and the rejection percentage (specificity) is the number of samples belonging to another class that are recognized as foreign to the model. The SIMCA model showed 100% recognition and rejection, indicating that the model identified all cocoa samples in their respective class and excluded all those from a different class with a 99% confidence level.

The SIMCA model was validated with external samples, and the results (Table 2) demonstrated the model’s ability to correctly identify samples from the three cocoa varieties. The statistical parameters are within the established limits (total distance ≤ 1, limit distance = 1, model distance = 0, and residual distance ≤ 3). This indicates that the model operates effectively and can be applied to identify samples of Criollo, Forastero, and Trinitario cocoa varieties with a 99% confidence level.

#### 3.3.3. Quantitative Models

Originally, an attempt was made to calibrate all nine parameters (moisture, protein, fat, ash, pH, acidity, phenolic compounds, ABTS, and DPPH) simultaneously. However, suitable results were not obtained due to certain spectral regions and pretreatments favoring some parameters while simultaneously affecting others. Therefore, based on PCA, those parameters with the highest correlation were selected and grouped into a single model. In the end, four predictive models were developed, grouped as follows: (1) moisture, pH, and acidity; (2) ash; (3) protein and fat; (4) phenolic compounds, ABTS, and DPPH in Table 3 present the spectral regions and pretreatments used in each model; these regions presented the best correlations between the analytical variations and the spectral response.

The four predictive models developed using the PLS algorithm (Table 4) showed satisfactory statistical results (7 < Factors < 12; 0.93 < R^2^c < 0.98; 0.03 < SEC < 4.34).

PLS calculates latent variables or factors from spectral data, which explain the maximum covariance between spectral data and analytical data. All four models presented factors between 7 and 12, and according to Beebe et al. [45], factors should be less than or equal to 50% of the number of samples used in the calibration set to avoid overfitting. R^2^c values ranged from 0.93 to 0.98, indicating that the variation of the actual value is accurately predicted in the calibration [22]. According to Tamaki and Mazza [46], R^2^c values above 0.90 describe quantitative information excellently. SEC values ranged from 0.03 to 4.34, indicating the precision with which the calibrated samples fit the regression [22]. The obtained statistical results demonstrate the ability of the four chemometric models to predict the chemical composition and antioxidant capacity of fermented dry cocoa beans.

This is further demonstrated in the external validation results (Table 3). R^2^v values were above 0.90 (0.93–0.97), indicating a good correlation between actual and predicted values. SEP values ranged from 0.04 to 3.59, indicating the error associated with the prediction. Additionally, this value helps assess the predictive capacity of the model when evaluating samples different from those used in the calibration [22]. Mahalanobis distance values were less than 1, and residual ratio values were less than 3, indicating that the cocoa samples used in the validation set have spectral similarity with the samples used in the calibration set. The percentage of error values was low, with the highest percentage obtained in Model 1 for acidity (10.35%). This can be attributed to the low acidity values determined in the samples; therefore, even a small variation between actual and predicted values results in a high percentage of error. The results obtained indicate that the four developed models provide accurate predictions. Therefore, these models were applied to five samples of cocoa beans different from those used in the calibration and validation stages.

**Figure 4:**
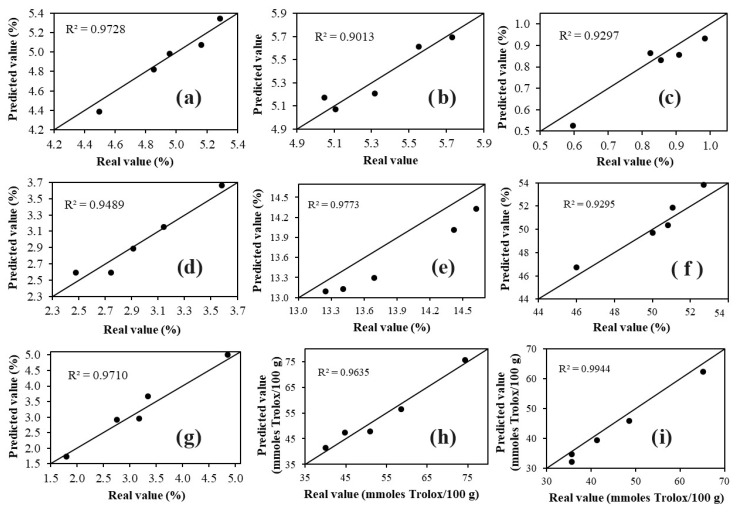
Plots of predicted values versus actual values of: (**a**) moisture, (**b**) pH, (**c**) acidity, (**d**) ash, (**e**) protein, (**f**) fat, (**g**) phenolic compounds, (**h**) ABTS, and (**i**) DPPH for the samples used to apply the model developed with PLS.

The results (Figure 4) confirmed that the four developed models successfully predicted the nine parameters (moisture, protein, fat, ash, pH, acidity, phenolic compounds, ABTS, and DPPH) in the external samples, as the predicted values were very close to those determined by conventional methods (R^2^ = 0.9013–0.9944). The results obtained from the application of the model (Figure 4) demonstrate that FT-MIR spectroscopy coupled with multivariate analysis yields similar results to those achieved with conventional techniques, but in a faster and more environmentally friendly manner, as it does not use reagents and solvents, unlike conventional methods that require a complex pretreatment before the analysis, are time-consuming, laborious, and use large amounts of reagents and solvents (noxious for the analyst and the environment) [9].

**Figure 5:**
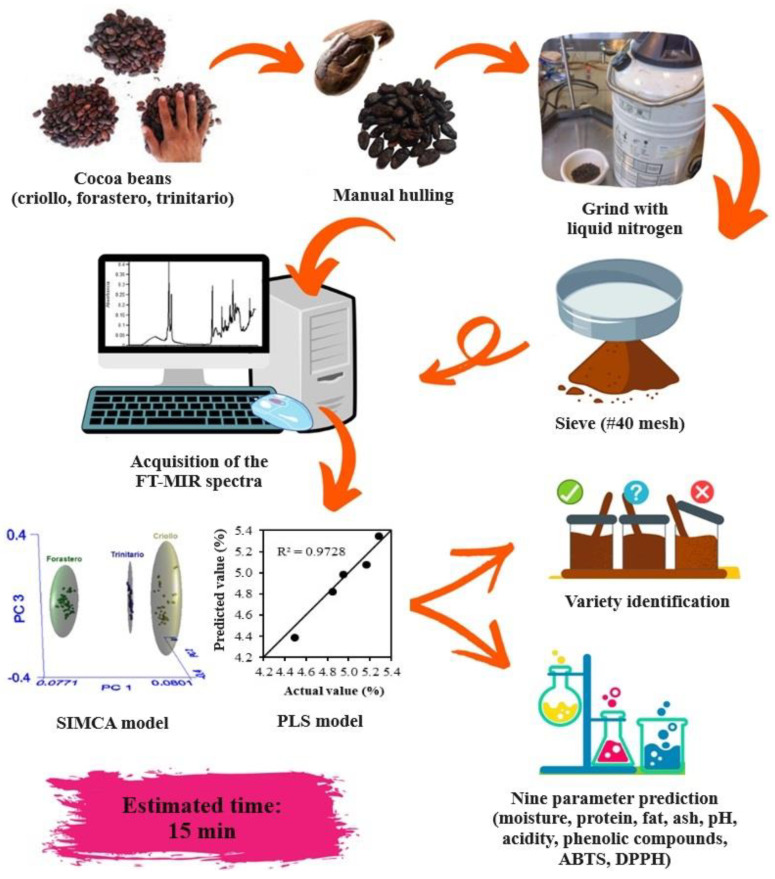
Flow diagram for the application of chemometric models.

Finally, in Figure 5, the application of the developed models to identify and quantify the chemical composition of cocoa beans is presented, with an estimated time of 15 min (considering sample preparation).

## 4. Conclusions

The developed models proved to be an alternative to conventional analysis, as they successfully identified the cocoa variety and accurately predicted their chemical composition in a maximum time of 15 min, using approximately 100 mg of sample. This is an advantage compared to conventional methods. In the future, calibration of other parameters relevant to quality control and other health-beneficial attributes is recommended.

## Figures and Tables

**Table 1:** Chemical analysis (moisture, protein, fat, ash, pH, acidity, phenolic compounds, and antioxidant capacity) of dry fermented cocoa beans.

	Criollo	Forastero	Trinitario
Moisture (%)	4.83 ± 0.62 ^b^	5.05 ± 0.49 ^a,b^	5.15 ± 0.49 ^a^
Protein (%)	13.24 ± 1.11 ^b^	14.44 ± 0.70 ^a^	14.45 ± 0.40 ^a^
Fat (%)	50.70 ± 2.97 ^a^	51.30 ± 1.09 ^a^	50.64 ± 1.68 ^a^
Ash (%)	3.13 ± 0.33 ^a^	3.12 ± 0.22 ^a^	3.24 ± 0.24 ^a^
pH	5.68 ± 0.58 ^b^	6.00 ± 0.48 ^a^	6.01 ± 0.41 ^a^
Acidity (% acetic acid)	0.65 ± 0.30 ^a^	0.69 ± 0.15 ^a^	0.62 ± 0.14 ^a^
Phenolic compounds (%)	3.96 ± 1.10 ^c^	5.66 ± 1.04 ^a^	4.74 ± 1.26 ^b^
ABTS (mmol TE/100 g dw)	49.83 ± 15.64 ^c^	90.88 ± 17.53 ^a^	78.87 ± 10.68 ^b^
DPPH (mmol TE/100 g dw)	47.03 ± 9.11 ^c^	66.88 ± 6.04 ^a^	60.32 ± 9.18 ^b^

Values represent means ± standard deviation. Means with different letters per row indicate significant statistical differences (Tukey, *p* ≤ 0.05).

**Table 2:** Validation results of the SIMCA model.

Samples	Identified Material ^a^	Result ^b^	Total Distance ^c^	Limit Distance ^d^	Model Distance ^e^	Residual Distance ^f^
**1–7**	Criollo	Identified	0.46–0.95	1.0	0.0	0.63–1.31
**1–10**	Forastero	Identified	0.51–0.99	1.0	0.0	0.65–1.27
**1–10**	Trinitario	Identified	0.61–0.93	1.0	0.0	0.81–1.23

^a^ Identified material by the SIMCA model; ^b^ result indicates if the sample was identified or rejected; ^c^ total distance must be less than 1; ^d^ limit distance must be equal to 1; ^e^ model distance must be equal to 0; ^f^ residual distance must be less than 3.

**Table 3:** Spectral regions and pretreatments applied in the models developed with the PLS1 algorithm.

Model	Spectral Regions (cm^−1^)	Pretreatments	
		Normalization	Baseline Correction
1	3700–3020, 1433–1279, 1225–1191, 977–725, 709–671, 663–550	Standard Normal Variate	First derivative, 2 points
2	3538–3027	Standard Normal Variate	Second derivative, 2 points
3	3450–2945, 1681–1578, 1450–1362, 1235–1189, 1169–724, 708–673, 663–550	Standard Normal Variate	Second derivative, 2 points
4	1720–1479, 1345–1190, 930–725, 715–673, 660–600	Standard Normal Variate	Second derivative, 2 points

**Table 4:** Calibration data to predict the chemical composition and antioxidant capacity of dry fermented cocoa beans.

		Calibration (*n* = 81)			Validation (*n* = 27)				
Calibration Set	Parameter	Factors ^a^	R^2^c ^b^	SEC ^c^	R^2^v ^d^	SEP ^e^	MD ^f^	RR ^g^	% Error ^h^
1	Moisture (%)	10	0.95	0.13	0.93	0.13	0.12–0.75	0.3–2.15	0.14–4.47
	pH	10	0.98	0.06	0.96	0.09	0.13–2.74	0.32–1.77	0.08–3.44
	Acidity (% acetic acid)	10	0.96	0.03	0.97	0.04	0.13–0.91	0.27–2.04	0.78–10.35
2	Ash (%)	10	0.93	0.06	0.93	0.07	0.13–0.97	0.66–2.11	0.45–5.05
3	Protein (%)	10	0.93	0.25	0.93	0.20	0.10–0.87	0.31–1.43	0.14–3.07
	Fat (%)	12	0.93	0.56	0.93	0.43	0.10–0.82	0.36–1.61	0.08–1.72
4	Phenolic compounds (%)	7	0.97	0.24	0.95	0.23	0.08–0.91	0.41–2.17	0.70–8.16
	ABTS (mmol TE/100 g dw)	8	0.95	4.34	0.95	3.59	0.08–0.73	0.39–2.39	0.31–9.58
	DPPH (mmol TE/100 g dw)	8	0.96	2.31	0.95	2.11	0.04–0.89	0.36–2.23	0.08–7.42

^a^ Factors; ^b^ R^2^c must be close to 1; ^c^ standard error of calibration should be as low as possible; ^d^ R^2^v must be close to 1; ^e^ standard error of prediction should be as low as possible; ^f^ Mahalanobis distance must be less than 1; ^g^ residual ratio must be less than 3; ^h^ % error should be as low as possible.

## Data Availability

Data used to support the findings of this study can be requested from the corresponding author.
